# Clinical Efficacy of Infantile Massage in the Treatment of Infant Functional Constipation: A Meta-Analysis

**DOI:** 10.3389/fpubh.2021.663581

**Published:** 2021-06-11

**Authors:** Zhi Liu, Li Gang, Ma Yunwei, Ling Lin

**Affiliations:** ^1^School of Precision Instrument and Opto-Electronics Engineering, Tianjin University, Tianjin, China; ^2^State Key Laboratory of Precision Measuring Technology and Instruments, Tianjin University, Tianjin, China; ^3^Office of Network and Security Information, Tianjin University of Traditional Chinese Medicine, Tianjin, China

**Keywords:** children's constipation, children's functional constipation, randomized controlled trials, Traditional Chinese Medicine, infant massage, meta-analysis

## Abstract

**Background:** Functional constipation in children is a common disease that causes a psychological burden on infants and young children across the world. It will greatly affect infant quality of life in early childhood and even affect their psychological and physical health. At present, infant functional constipation is treated with western drugs alone, but this can produce drug dependency. In recent years, Traditional Chinese Medicine (TCM) infant massage has been used as a complementary and alternative therapy, and its effectiveness and safety have been proven, attracting the attention of numerous researchers.

**Objective:** Our study aimed to compare the influence of infant massage intervention on defecation frequency and consistency, determine the effectiveness, and safety of infant massage in the treatment of infant functional constipation, and obtain high-quality clinical evidence.

**Methods:** Based on the Preferred Reporting Items for Systematic reviews and Meta-Analyses (PRISMA) Statement, inclusion, and exclusion criteria were formulated. Randomized controlled trials (RCTs) on TCM infant massage for the treatment of infant functional constipation were found following a search of four mainstream medical databases. RCTs found to meet the study's requirement were included; data information was then extracted, and the quality was assessed using the Cochrane bias risk assessment tool. Through RevMan software, a meta-analysis was carried out for overall effective rate, stool form, defecation frequency, defecation difficulty, and constipation symptom scoring index. The relative risk (RR) and 95% confidence interval (95% CI) were calculated, heterogeneity was tested and its source was found, and publication bias was assessed through the Egger's and Begg's tests and by means of funnel plots.

**Results:** A total of 23 RCTs and 2,005 patients were included. The results of the meta-analysis showed that compared to drug therapy alone, TCM infant massage had a superior effect on the treatment of infant functional constipation. This difference was statistically significant (*p* < 0.05) and evaluated according to the overall effective rate (RR = 1.25; 95% CI = 1.17, 1.33), defecation frequency [mean difference (MD) = −0.72; 95% CI = −0.80, −0.65], and constipation symptom score (MD = −0.81; 95% CI = −1.20, −0.43), showing that TCM infant massage is indeed superior to drug therapy alone in the treatment of infant functional constipation. TCM infant massage was found to be equivalent to drug therapy alone in terms of the stool form score [−0.30 (−0.38, −0.22)] and the defecation difficulty score [−0.73 (−0.81, −0.65)], since the difference was not statistically significant (*p* > 0.05). The source of heterogeneity might be related to the state of patient, manipulation of the massages, efficacy of drugs in the control group, and difference in judgment criteria for efficacy. The Egger's test and Begg's test showed that publication bias did not occur in our study.

**Conclusion:** TCM infant massage can increase defecation frequency and reduce the symptoms of constipation in children suffering from functional constipation; in addition, the clinical trial showed beneficial effects. Since some of the RCTs featured a very small sample size, the reliability and validity of our study's conclusion may have been affected as well; therefore, the explanation should be treated with some caution. In the future, a large number of higher-quality RCTs are still needed to confirm the results of our study.

## Introduction

Infant functional constipation is a common disease in childhood; it has an incidence rate of 0.3–8%, accounting for 3–5% of pediatric outpatients (1). Its prevalence rate is 0.7–29.6% in the field of pediatric health care across the world, including both developed and developing countries (2). In China, there are relatively few epidemiological studies on this disease. The condition has adverse effects on infant sleep and appetite and even endangers their growth and development (3). It affects children's quality of life. Compared to healthy children, those with constipation were found to have a lower score of quality of life, far lower than even the impact score mentioned in studies related to gastroesophageal reflux disease (GERD) and inflammatory bowel disease (IBD) (4). In addition, a heavy economic burden is brought by this disease (5); the average annual expenditure for treating children with constipation are three times higher than those without this disease (6). Moreover, some diseases such as headache, depression, anxiety, influenza, otitis media, and asthma are more common in children with constipation, which will further increase their medical expenses (7).

According to the recommendations of the North American Society for Pediatric Gastroenterology, Hepatology & Nutrition, therapies for constipation in children usually include family education, diet changes, potty training, use of laxatives and other drugs, and behavioral changes (8). Among the existing treatment options, drug therapy is still the first choice. The most commonly used laxatives for children with functional constipation are lubricants and penetrants (9). Osmotic laxatives are the most popular drugs, and polyethylene glycol (PEG) has become a successful choice for laxative treatment (10). Drugs for infant functional constipation have a certain effect on alleviating the symptoms of constipation, but they take a long time, usually 3–6 months; in addition, the overall curative effect is not satisfactory, with a high recurrence rate (11). It has been proven that only 60% of children have no symptoms 6–12 months after treatment (12). In longer follow-ups, one of four of children still have symptoms, and in some cases, the symptoms continue into adulthood (11). Therefore, both study and assessment are urgently required to identify more effective treatment regimens for infantile constipation.

Due to the limitations of current standard care, investigators are searching other methods from complementary and alternative medicine (CAM) therapies. About 24.1% of children with functional constipation received CAM therapies; 93% of parents believed that clinical studies were necessary for CAM, and only 51% agreed for their children to participate in such studies (13). Aside from parents' views, it is realistic, and even urgent, to evaluate the effectiveness and safety of CAM therapies in children. Massage therapy has a long history of use as a traditional diagnosis/treatment method within CAM therapy and is an important component of Traditional Chinese Medicine (TCM). Infantile massage is a subset of TCM massage and is the result of historic advances for patients in certain states of development; it emerged as a result of traditional practices. The first records of infantile massage can be found in “Folk Recipes for Infantile Disease and Proven Recipes for Infants in the Prescriptions for Fifty-Two Diseases” (a medical book copied on silk that was unearthed from the Mawangdui Tomb, dated to the Western Han Dynasty). TCM is based on the following concepts: by exerting a certain stimulation on the surface of an infant's body, the technique is used to dredge channels, collaterals, qi, and blood and thereby to prevent and treat diseases. Moreover, since the viscera of infants are traditionally viewed as “clear and bright” in terms of physiological characteristics and show a near-instantaneous response to pinching, a high efficacy can be achieved for infants *via* massage. Infantile massage is a common method of physical therapy, partly as it is highly environmentally friendly, free of toxic chemicals or side effects, and therefore likely to be accepted by the infants' parents or family (14).

In this systematic review and meta-analysis, TCM infant massage was selected as a representative CAM therapy to treat infant functional constipation and evaluate its efficacy and safety in the treatment of constipation in children.

## Methods

### Searches

Four mainstream medical databases were searched, including PubMed, China Network Knowledge Infrastructure (CNKI), WanFang, and China Science and Technology Journal Database (CSTJ). The time frame used for database queries was from the earliest indexed studies to March 19, 2020. Search words included “Tuina” (Chinese for “massage”), massage, manipulation, chiropractic, spinal manipulation, children, infantile, and child constipation. In order to ensure the completeness of the search, the language of the database was not restricted; a search was carried out through a combination of MeSH keyword and free words; the retrieval strategy could thus be finalized (the concrete retrieval formula of the database is shown in Appendix). Gray literature was manually retrieved for supplementation at the library of the Tianjin University of Traditional Chinese Medicine; if the full texts were not available, a mail was sent to its author in an attempt to obtain the full document.

### Inclusion Criteria

#### Type of Studies

Randomized controlled trials (RCTs), with no language restrictions.

#### Subjects

Children diagnosed with functional constipation according to the diagnostic criteria were not restricted in terms of gender, source of cases, and age. The diagnostic criteria for functional constipation included Rome III Criteria (15), Interim Standards for Diagnosis and Treatment of Constipation formulated by the editorial board of the Chinese Medical Journal (16), Pediatrics of Chinese Medicine (17), Diagnostic Efficacy Standard of TCM Diseases and Symptoms (18) and Guiding Principles for Clinical Research of New Chinese Medicines (19) formulated by the NATCM, and Constipation Symptoms and Efficacy Evaluation (20) formulated by the Anorectal Surgery Group of the Chinese Medical Association Surgery Branch.

#### Intervention Measures

In the treatment group, massage therapy combined with basic treatment, or combined with other therapies (without limitation on the techniques and positions of massage), was adopted; in the control group, basic treatment or other therapies were adopted, with no massage therapy (the basic treatment or other therapies were the same in the two groups).

#### Evaluation Indices

Evaluation indices included overall effective rate, stool form score, defecation difficulty score, defecation frequency, and constipation symptom score. Overall effective rate was evaluated based on the assessment criteria for efficacy of infant functional constipation in the following documents: Guideline for Clinical Trial on New Drugs of Chinese Medicines, Tentative Criteria for Diagnosis and Treatment of Constipation (Group of Anus & Intestine Surgery, Society of Surgery, Chinese Medical Association), or Assessment of Symptoms and Efficacy of Constipation (Group of Anus & Intestine Surgery, Society of Surgery, Chinese Medical Association), or Criteria for Diagnosis and Efficacy of Syndromes of Chinese Medicine. Stool form score was assessed through stool form assessment table (Bristol); defecation difficulty score was evaluated through constipation assessment scale (CAS); defecation frequency was assessed through complete spontaneous bowel movements (CSBMs); constipation symptom score was evaluated through patient assessment of their constipation symptoms (PAC-SYM).

### Exclusion Criteria

(1) Studies without random grouping; (2) studies that provided incomplete information, no correct data could be obtained even after contacting the authors and through data calculations, etc.; (3) studies without control groups; (4) studies with obvious data errors; (5) duplicated studies; (6) papers of non-RCTs, such as review, clinical experience introduction, summary analysis, clinical case reports, literature involving massage genre, animal experiments; (7) studies with control group treatment of infant massage; (8) studies that did not use constipation as a research object; (9) literature on nursing.

### Data Selection and Data Extraction

**Figure 1:**
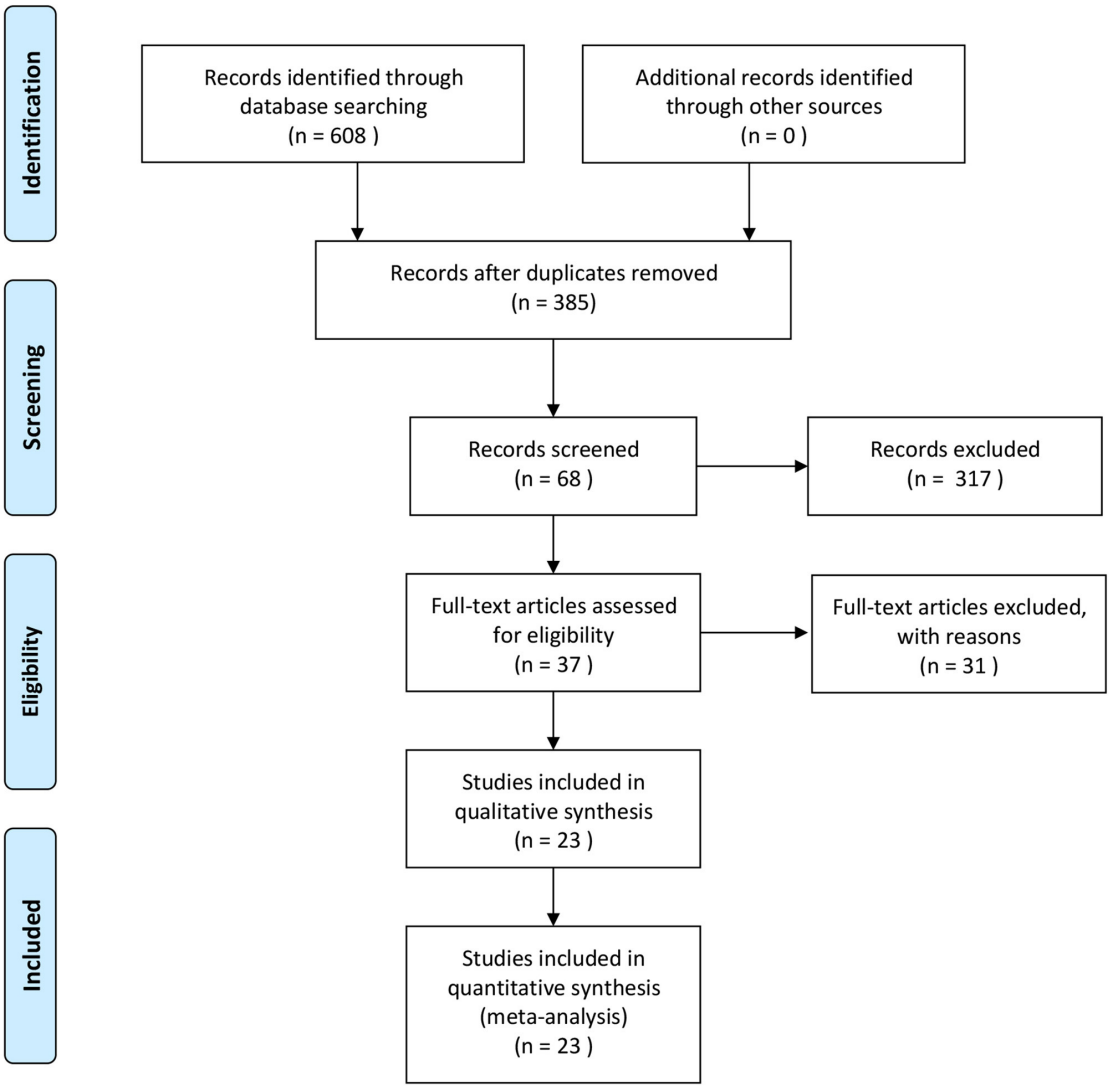
Study screening flowchart. PRISMA, preferred reporting items for systematic reviews and meta-analyses.

First, all literature was screened using End-Note 9.0 software according to the preset inclusion/exclusion criteria, and any duplicated literature from different databases was removed. Then, any obviously irrelevant literature was excluded based on the title and abstract. Finally, the full text of all remaining literature was read intensively, and those papers conforming to the requirements were included (see Figure 1: Study flowchart). The data were extracted independently by two investigators; a cross-check was made. Any disagreement was settled through discussion or the arbitration of a third investigator. The extracted data included the name of the first author, year of publication, sample size, average age and gender of participants, interventions of treatment group and control group, duration of treatment, prognostic indicators, and a literature quality evaluation. Adverse events were tabulated to record the occurrence in the test group and control group. Adverse events included intestinal reactions, allergic reactions, mental fatigue, and poor sleep.

### Quality Evaluation

The quality of studies included was evaluated with reference to the quality evaluation criteria recommended by the Cochrane System Evaluator Manual Version 4.2.2. The contents of this evaluation included generation of random sequence allocation concealment, investigator/subject blinding, blind-method evaluation of study outcome, integrity of outcome data, selective reporting of study results, and source of other biases. Two evaluators would conduct independent evaluations based on uniform quality standards, and if they found any discrepancy, they would resolve them through discussion; if it still could not be solved, a third investigator would be asked to reach a consensus. Using the RevMan software, the bias risk assessment chart and bias risk assessment table were exported to show evaluation results for all included literature.

### Statistical Analysis

Excel tables were made for the data extraction. The differences in outcome measures before and after treatment in the treatment group and the control group were calculated, and two evaluators would input the data for the differences between the treatment group and the control group, and their standard deviations into RevMan5.3 software independently, to perform the meta-analysis. The count data were expressed as a relative risk (RR), measurement data were expressed as a mean difference (MD), and the interval estimation used a 95% confidence interval (95% CI). *p* < 0.05 indicates that the difference was statistically significant. *Q* test and *I*^2^ value were used to analyze the heterogeneity among the included studies. *p* > 0.1 and *I*^2^ < 50% indicated a good consistency or lower heterogeneity among included studies, and a fixed-effects model was adopted; otherwise, a random-effects model was used. *I*^2^ ≤ 25% indicated a smaller heterogeneity; 25% < *I*^2^ ≤ 50% indicated a moderate heterogeneity, both of which could be acceptable. *I*^2^ > 50% indicated high heterogeneity between the results of the study. For higher heterogeneity, a subgroup analysis was made to explore the source of this heterogeneity; when necessary, a sensitivity analysis was made to assess the stability of the study. Through the Egger's test and the Begg's test carried out in the Stata software and the funnel chart, publication bias was tested. As required by the index, more than 10 pieces of literature should be included, since it would be difficult to find the reason for asymmetry if there were too few studies.

## Results

### Results of Literature Retrieval

A total of 608 pieces of literature were obtained through database retrieval, though no other routes, such as manual retrieval, were taken. There were 480 Chinese papers and 128 English papers. After the elimination of duplicate literature, 385 papers were obtained. After the reading of title and abstract, 317 pieces of literature not conforming to the requirement were removed; the remaining 37 papers entered the stage of full-text reading. Finally, a total of 23 articles on RCTs were included in our study (21–43).

### Basic Characteristics of the Studies Included

As shown in Table 1, 23 studies involving 2,005 patients (including 1,006 subjects in the test group and 999 subjects in the control group) were analyzed. In our study, the sample size ranged from 40 to 122. The time span for literature publication was from 2010 to 2020. All studies were carried out in China. Only five studies (22, 24, 35, 36, 38) reported adverse events, and no subsequent studies were mentioned. General information on the subjects was lacking in six articles (26, 29, 31, 37, 39, 40), and there was no statistically significant difference in baseline in 21 articles (20–30, 32–40, 42). The basic characteristics of these articles are shown in Table 1.

**Table 1:** Basic characteristics of the included literature.

**References**	**Sample size**	**Number of cases (M/F)**		**Age (years)**		**Interventions**		**Course of treatment (days)**	**Evaluation indexes**
		**T**	**C**	**T**	**C**	**T**	**C**		
Sun (21)	64	16/16	14/18	2.68 ± 1.90	2.64 ± 1.83	Infant massage	Wangshi Baochi Pills, Oral	12	①
Li (22)	76	12/26	16/22	2.10 ± 1.21	1.95 ± 1.14	Infant massage	Live Combined *Bifidobacterium, Lactobacillus*, and *Enterococcus* Capsules, Oral	14	①②③④
Chen and Wang (23)	60	14/16	12/18	2.27 ± 1.48	2.10 ± 1.44	Infant massage	Medilac-Vita, Oral	14	① ②③④
Huang (24)	80	22/18	21/19	1.80 ± 0.24	1.98 ± 1.17	Infant massage	Conventional medication	60	①
Chen et al. (25)	120	33/27	35/25	2.8 ± 1.2	2.9 ± 1.5	Infant massage	Medilac-Vita, Oral	14	①②④
Jia (26)	72	19/17	20/16	5.88 ± 1.62	5.91 ± 1.59	Infant massage + Simotang oral liquid and Medilac-Vita	Simotang oral liquid and oral Medilac-Vita	14	①
Hao et al. (27)	72	19/17	20/16			Infant massage	Medilac-Vita, Oral	10~20	①
Xu (28)	122	30/31	32/29	7.24 ± 2.51	7.41 ± 2.46	infant massage+ oral Chinese medicines	Oral Chinese medicines alone	14	①
Xu (29)	40	10/10	9/11	5.35 ± 4.17	5.58 ± 4.39	Infant massage	Chinese medicine prescription	5~10	①
Shen et al. (30)	106	32/21	28/25			Infant massage + Oral Zengye Decoction	TCM Decoction-Oral Zengye Decoction	20	①
Wang (31)	90	21/24	23/22	3.3 ± 0.8	3.1 ± 0.6	Infant massage	Medilac-Vita, oral	10	①
Wang et al. (32)	105					Infant massage + Probiotic treatment	Live Combined *Bifidobacterium, Lactobacillus*, and *Enterococcus* Capsules, oral	未提及	②
Wang (33)	120	25/35	22/38	5.53 ± 0.78	5.84 ± 0.62	Infant massage + Baohe Pills	Baohe Pills, Oral	7	①
Mao (34)	94	25/22	24/23	7.51 ± 3.16	7.43 ± 3.08	Infant massage + standard care	Qidaozhi Tongfu Recipe+standard care	14	②
Kang (35)	78	21/18	16/23	1.18 ± 1.15	1.37 ± 1.62	Infant massage + standard care	Live Combined *Bifidobacterium, Lactobacillus*, and *Enterococcus* Capsules +standard care	7~35	③
Zhang (36)	84	23/19	25/17	1.54 ± 0.39	1.48 ± 0.37	Infant massage + Medilac-Vita	Medilac-Vita	14	①
Cao (37)	70	16/19	18/17	1.40 ± 0.13	1.43 ± 0.10	Infant massage	Siliankang	14	①
Zhu and Huang (38)	116	32/28	30/26			Infant massage + Siliankang and standard care	Siliankang and standard care	30	①
Li and Lin (39)	80	23/17	22/18	6.69 ± 3.74	6.58 ± 3.88	Infant massage + Medilac-Vita and routine care	Medilac-Vita and routine care	5	①
Li et al. (40)	90	22/23	23/22	3.6 ± 0.5	3.5 ± 0.7	Infant massage	Xiaoer Kaiwei Zengshi Mixture, Oral	21	①
Li et al. (41)	86	24/22	22/18			Infant massage	Medilac-Vita, Oral	5~10	①
Yu et al. (42)	80	21/19	18/22			Infant massage	Retention enema with kaiselu	3~6	①
Liu and Yang (43)	100	31/19	29/21	3.02 ± 0.82	2.92 ± 0.85	Infant massage	Medilac-Vita, Oral	10	①

*Evaluation indicators: Efficacy; Stool form; Defecation frequency; Defecation difficulty; Score of constipation symptoms*.

### Quality Evaluation of Included Studies

**Figure 2:**
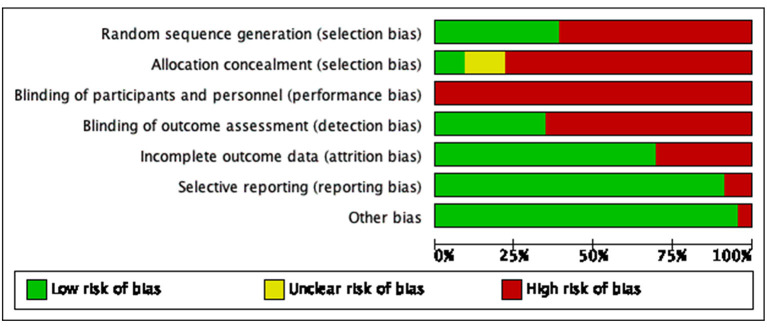
Bias risk diagram: judgment of risk of bias expressed as a percentage of all included studies.

**Figure 3:**
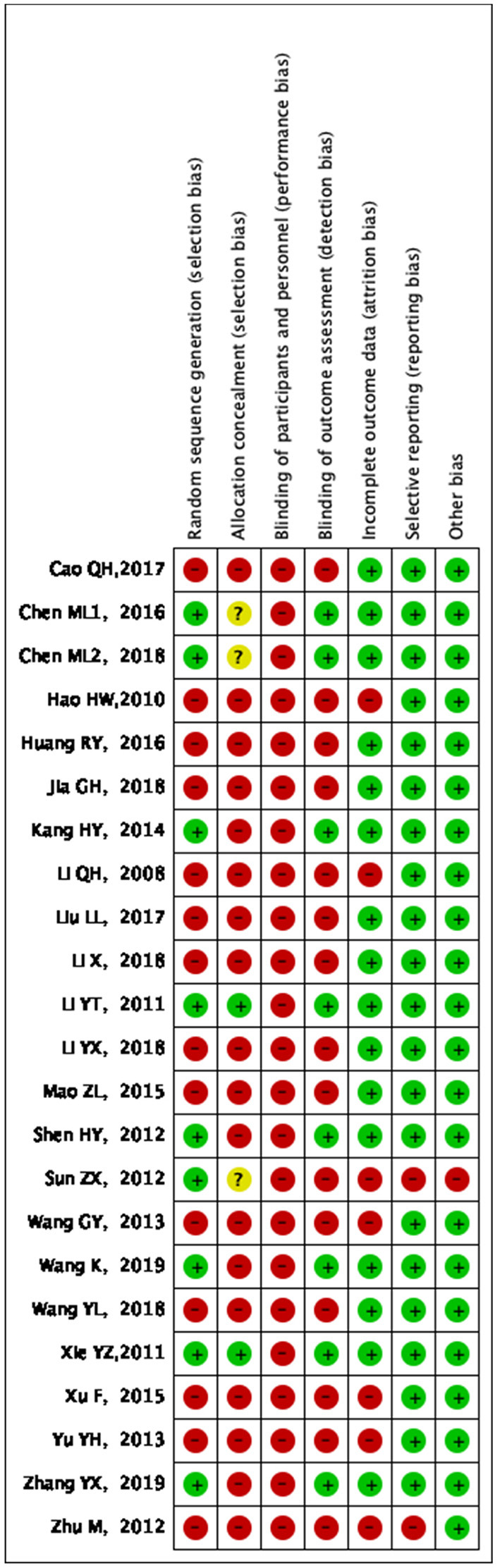
Bias risk summary: judgment of risk of bias and items with bias included in the studies; “+” = low risk, “–” = high risk, “?” = unclear.

Generation of random sequences: Random number tables were used for random grouping in eight studies (20–22, 24, 29, 32, 34, 35), random envelopes were used for random allocation in one study (28); visiting time was used for randomization in one study (27), visiting sequence was used for random allocation in one study (31), and other studies only mentioned the word “randomization.” Allocation concealment: A total of two studies (21, 28) used allocation concealment. Investigator/subject blinding: None of the studies used the blinding method. Selective reporting of study results: Follow-up was reported in three studies (21, 23, 38). Integrity of outcome data: No criteria for termination, rejection, or withdrawal were established in any of the included studies, and no dropouts were reported. Detailed results of bias risk assessment are summarized in Figures 2, 3.

### Results of Meta-Analysis

#### Analysis of Effective Rate

**Figure 4:**
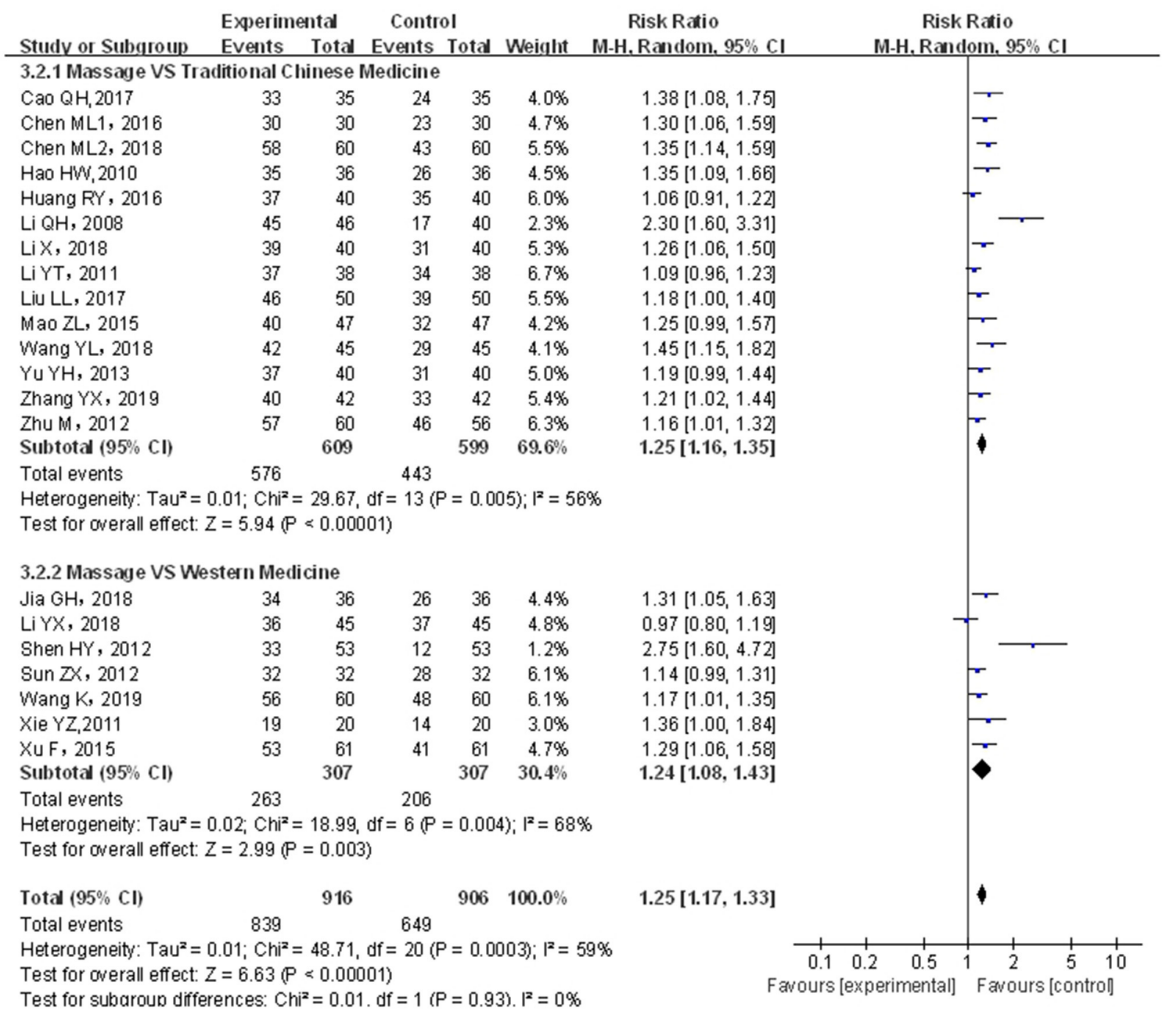
Meta-analysis of infant massage and drug therapy in the treatment of children with functional constipation.

A total of 21 papers covering 1,822 patients were included. Analytical results are shown in Figure 4; the included studies were of significant heterogeneity (*p* < 0.05, *I*^2^ = 59%); using the random-effects model, where statistics were combined. As compared with traditional drug therapy alone, the combination with infantile massage had a higher effective rate for infant functional constipation (RR = 1.25; 95% CI = 1.17, 1.33), where the difference was statistically significant (*p* < 0.05). Since heterogeneity was higher, which might be related to the type of drug used in the control group, a subgroup analysis was carried out based on classification into Chinese medicine and western medicine. In the control group, the degree of heterogeneity was still higher for Chinese medicine (*p* < 0.05, *I*^2^ = 56%) and western medicine (*p* < 0.05, *I*^2^ = 68%), which did not change significantly; therefore, different types of drug in the control group was not the source of heterogeneity. In our view, higher heterogeneity might be related to multiple factors, such as different illnesses of subjects included in each study, different manipulation of massage in the treatment group, and different judgment criteria for efficacy.

#### Scoring of Stool Form

**Figure 5:**
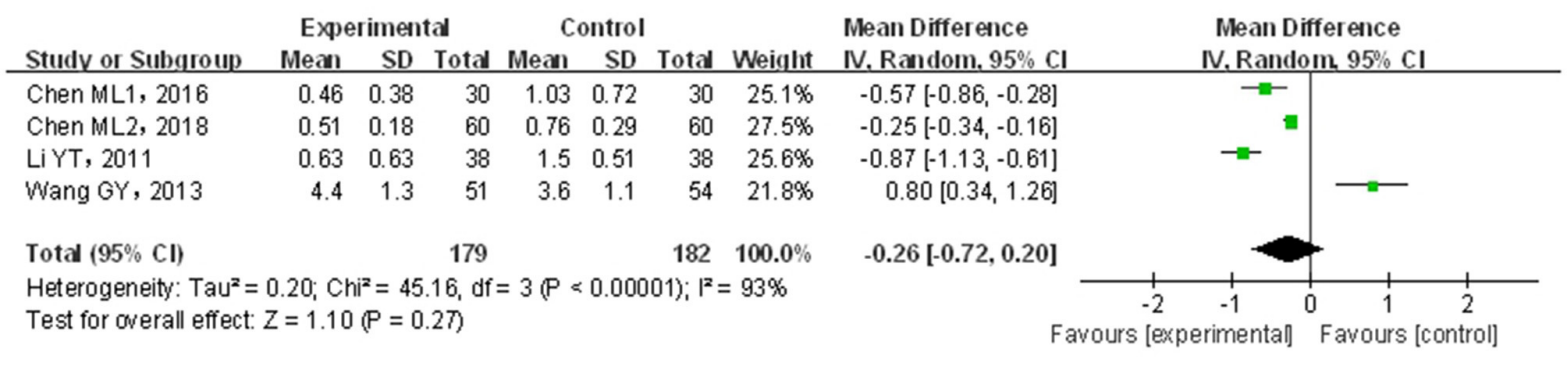
Meta-analysis of infant massage and drug therapy in the treatment of children with functional constipation to reduce stool form scores.

A total of four papers (21, 22, 24, 31) covering 361 patients were included. Results are shown in Figure 5. The included studies were of significant heterogeneity (*p* < 0.05, *I*^2^ = 95%); through the random-effects model, the statistics were combined. As compared with traditional drug therapy alone, the combination with infantile massage had an equivalent efficacy in stool form score (MD = −0.26; 95% CI = −0.72, 0.20), where the difference was not statistically significant (*p* > 0.05). Since the heterogeneity was higher, the results of the sensitivity analysis showed that the outcome of the disease was influenced by intervention measures, which were not consistent with overall results (see the Appendix). Therefore, the included studies were of larger overall influence and displayed unstable results.

#### Defecation Frequency

**Figure 6:**
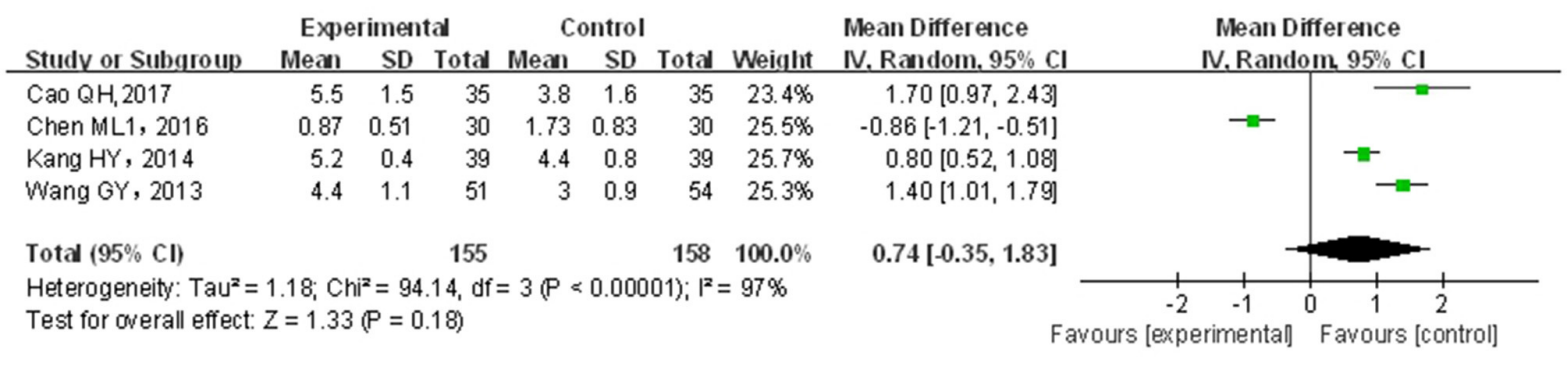
Meta-analysis of infant massage and drug therapy in the treatment of children with functional constipation on change in defecation frequency.

A total of four papers (22, 31, 34, 36) covering 313 patients were included. Results are shown in Figure 6. The included studies were of significant heterogeneity (*p* < 0.05, *I*^2^ = 97%); through the random-effects model, statistics were combined. Compared with traditional drug therapy alone, the combination with infantile massage showed an equivalent efficacy in the increase of defecation frequency (MD = −0.74; 95% CI = −0.35, 1.83), where the difference was not statistically significant (*p* > 0.05). Since the heterogeneity was higher, the results of the sensitivity analysis showed that the outcome of the disease was not influenced by intervention measures, which was not consistent with overall results. The reason for heterogeneity increase could not be screened out. In our view, the higher heterogeneity might be related to multiple factors, such as sample size, different illnesses of subjects included in each study, different manipulation methods for massage in the treatment group, and different judgment criteria for efficacy.

#### Defecation Difficulty Score

**Figure 7:**
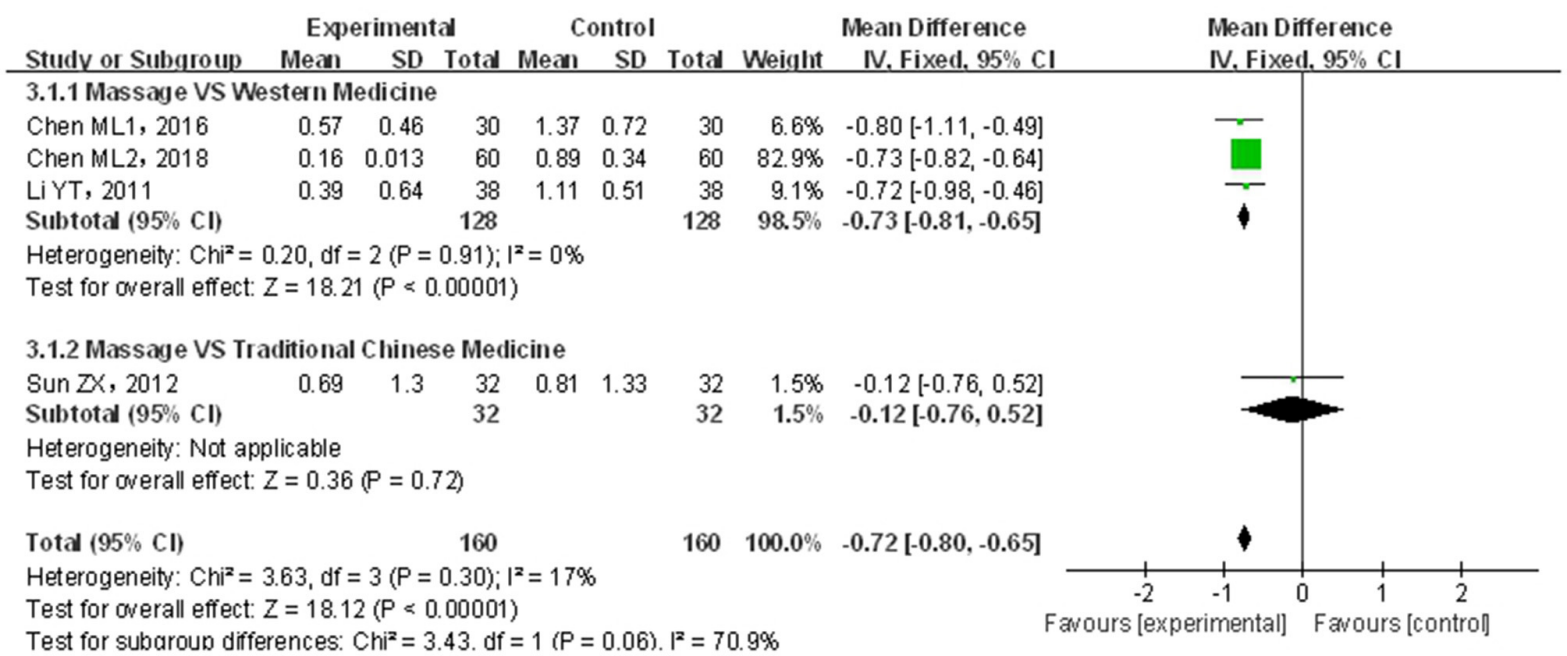
Meta-analysis of infant massage and drug therapy in the treatment of children with functional constipation to reduce defecation difficulty score.

A total of four papers (20–22, 24) covering 320 patients were included. Results are shown in Figure 7. The included studies were not of significant heterogeneity (*p* > 0.05, *I*^2^ = 17%); using the fixed-effects model, the statistics were combined. As compared with traditional drug therapy alone, the combination with infantile massage had a higher score for improving defecation difficulty (MD = −0.72; 95% CI = −0.80, −0.65), where the difference was statistically significant (*p* < 0.05). Since the heterogeneity of the included studies was better, a subgroup analysis was not required to explore the source of this heterogeneity.

#### Constipation Symptom Score

**Figure 8:**
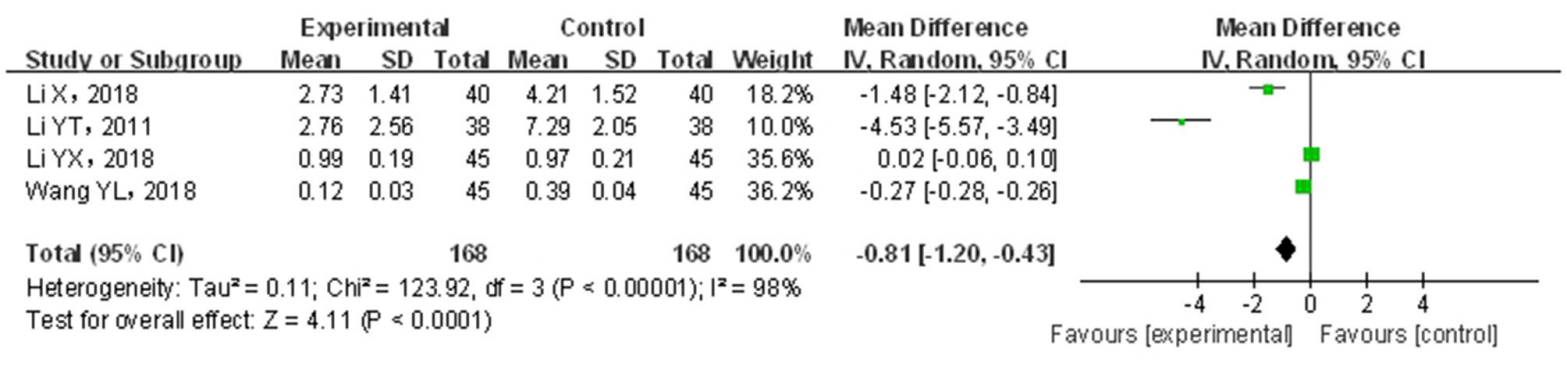
Meta-analysis of infant massage and drug therapy in the treatment of children with functional constipation to reduce constipation symptom score.

A total of four papers (21, 30, 38, 39) covering 336 patients were included. Results are shown in Figure 8. The included studies showed significant heterogeneity (*p* < 0.05, *I*^2^ = 98%); through the random-effects model, the statistics were combined. Compared with traditional drug therapy alone, the combination with infantile massage was more superior in reducing constipation symptom scores (MD = −0.81; 95% CI = −1.20, −0.43), where the difference was statistically significant (*p* < 0.05). Since the heterogeneity was higher, the results of the sensitivity analysis showed that the outcome of the disease was influenced by intervention measures, which was consistent with overall results (see the Appendix). The reason for heterogeneity increase could not be screened out. In our view, higher heterogeneity might be related to multiple factors, such as sample size, different illnesses of subjects included in each study, different manipulation techniques in the massage treatment group, and different judgment criteria for efficacy.

#### Adverse Events

Among the included literatures, five papers (22, 24, 35, 36, 38) mentioned adverse events. Four of these five saw no adverse event reported in either of their research groups, while in the remaining article (38), 22 cases of adverse events were reported, including three cases in the test group and 19 cases in the control group. The number of cases with adverse events in the test group was less than that in the control group.

#### Analysis of Publication Bias

Publication bias was analyzed by means of a funnel chart. After combination with infantile massage, the included studies showed no symmetric distribution in terms of overall effective rate, indicating that there was a publication bias. As shown by the results of the statistical analysis, *Pr*>^|z|^ index was 0.401 in the Egger's test and *p* > *t* index was 0.420 in the Begg's test, indicating that there was no publication bias based on the statistical tests.

## Discussion

As found by clinical trials on infant constipation, infantile functional constipation refers to constipation that is not caused by organic factors. Defecation frequency in healthy babies and children decreases with age. Breastfeeding children may defecate as much as 12 times a day, or in very rare cases, only once every 3–4 weeks. After infants are fed with milk powder, their stools are generally formed at Week 1. Infants can frequently experience pain during defecation and develop functional constipation. As an *in vitro* method of physical diagnosis/treatment, massage is not only effective but also more able to ensure the safety of children with functional constipation; it alleviates or eliminates the dependence of such children on drug therapy alone and eliminates the risk of side effects from drugs. It thus better promotes their growth and should be strongly recommended in future guidelines for clinical practical methods.

At present, clinical trial methods for various massages (including TCM infantile spinal pinching) are not of high quality, and clinical evidence is weak. On this basis, a meta-analysis was carried out in our study. Compared with conventional drug therapy, a combination with infantile massage was definitely superior for the treatment of children with functional constipation in terms of effective rate, defecation difficulty score, and constipation symptom score, where the difference was statistically significant. However, since the included studies were of lower quality and higher heterogeneity, these results should be treated with a certain amount of caution. In recent years, numerous studies on CAM therapy for infantile constipation have been published. For example, in a study of meta-analysis of acupuncture combined with Chinese medicine for functional constipation (44), this combined therapy was effective and safe for functional constipation and could improve the constipation symptom score. Compared with this study, our study possessed two features. (1) Since the age of the children studied was younger, massage combined with drug therapy was of higher clinical safety and more suitable for clinical popularization than acupuncture combined with drug therapy. (2) Our study involved more literature review (203 vs. 608). However, publication bias was the main reason for influencing the validity of results of the meta-analysis because it was the main basis for making a conclusion to obtain the published studies. Moreover, positive results were overemphasized by some editors or in some magazines, so that some negative results were concealed. Meanwhile, a loss of literature could be caused by an insufficient retrieval method and various other reasons. In our study, the analysis of the effective rate showed no publication bias; however, since four other indices were limited by too small a sample size, the corresponding analysis was not made.

This systematic evaluation and meta-analysis were performed strictly according to the Preferred Reporting Items for Systematic reviews and Meta-Analyses (PRISMA) Statement: it first evaluated the clinical efficacy and safety of infantile massage for infant functional constipation; it evaluated how standard the clinical trial was from a methodological point of view and also offered a direction for further study. However, it is important to note that our study still had certain limitations. (1) The included literature was of low quality; the generation of random allocation sequences and the concealment of randomization protocols were not reported in some studies; the conditions of dropout and withdrawal from the study were not described in detail; certain problems occurred (such as missing study data), which influenced the evidence level and popularization degree. (2) Cases were not fully included according to generally recognized international diagnostic criteria; standard uniform outcome measurement indices were not observed; a subjective composite outcome scoring index was adopted, and the evaluation criteria for efficacy were formulated by ourselves. (3) Treatment courses were shorter; there were no important clinical indices for long-time observation, and the superiority of infantile massage in terms of overall efficacy could not be fully displayed.

## Conclusion

At present, infantile massage is a safer, more effective method of CAM therapy for infant functional constipation. It has various advantages, such as high clinical effectiveness, improvement of constipation symptoms, and alleviation of defecation difficulty. It is worthy of clinical popularization and application. Since the original studies were of too low quality, a strict standard clinical trial should be made for verification in the future under the premise that the TCM characteristics of infantile massage are ensured.

## Data Availability Statement

The datasets presented in this study can be found in online repositories. The names of the repository/repositories and accession number(s) can be found in the article/Supplementary Material.

## Author Contributions

ZL and LG took part in the design of the study, performed the literature survey, and drafted the manuscript. MY took part in data management implementation of the study. ZL was responsible for the statistical analysis and methodological design of the study. All the authors read and approved the final manuscript and have made substantive contributions to this study in regard to design and implementation.

## Conflict of Interest

The authors declare that the research was conducted in the absence of any commercial or financial relationships that could be construed as a potential conflict of interest.
