# Supplementary material for: Clinical Efficacy of Infantile Massage in the Treatment of Infant Functional Constipation: A Meta-Analysis
Source: Front Public Health. 2021 Jun 11;9:663581. doi: 10.3389/fpubh.2021.663581 (PMC8232057; doi:10.3389/fpubh.2021.663581)
Supplement: Supplementary file 1 [file Data_Sheet_1.pdf]

Appendix:

Table 1 Basic characteristics of the included literature

Author and publication year	Sample size	Number of cases (M/F)		Age (years)		Interventions		Course of treatment (days)	Evaluation indexes
		T	C	T	C	T	C		
Sun Zhengxin [i], 2012	64	16/16	14/18	2.68±1.90	2.64±1.83	Infant massage	Wangshi Baochi Pills, Oral	12	①
Li Yuting [ii], 2011	76	12/26	16/22	2.10±1.21	1.95±1.14	Infant massage	Live Combined Bifidobacterium, Lactobacillus and Enterococcus Capsules, Oral	14	①②④⑤
Chen Meilan 1[iii], 2016	60	14/16	12/18	2.27±1.48	2.10±1.44	Infant massage	Medilac-Vita, Oral	14	① ②③④
Huang Ruiyi [iv], 2016	80	22/18	21/19	1.80±0.24	1.98±1.17	Infant massage	Conventional medication	60	①
Chen Meilan 2 [v], 2018	120	33/27	35/25	2.8±1.2	2.9±1.5	Infant massage	Medilac-Vita, Oral	14	①②④
Jia Guohua[vi], 2018	72	19/17	20/16	5.88±1.62	5.91±1.59	Infant massage + Simotang oral liquid and Medilac-Vita	Simotang oral liquid and oral Medilac-Vita	14	①
Hao Hongwen[vii],2010	72	19/17	20/16			Infant massage	Medilac-Vita, Oral	10~20	①
Xu Fen c[viii], 2015	122	30/31	32/29	7.24±2.51	7.41±2.46	infant massage+ oral Chinese medicines	Oral Chinese medicines alone	14	①
Xu Fen [ix],2011	40	10/10	9/11	5.35±4.17	5.58±4.39	Infant massage	Chinese medicine prescription	5~10	①
Shen Hongyan[x], 2012	106	32/21	28/25			Infant massage + Oral Zengye Decoction	TCM Decoction-Oral Zengye Decoction	20	①
Wang Yali [xi], 2018	90	21/24	23/22	3.3±0.8	3.1±0.6	Infant massage	Medilac-Vita, oral	10	①

Wang Guoyi [xii], 2013	105					Infant massage + Probiotic treatment	Live Combined Bifidobacterium,Lactobacillus and Enterococcus Capsules , oral	未提及	②
Wang Ke[xiii], 2019	120	25/35	22/38	5.53±0.78	5.84±0.62	Infant massage + Baohe Pills	Baohe Pills, Oral	7	①
Mao Zhilong [xiv], 2015	94	25/22	24/23	7.51±3.16	7.43±3.08	Infant massage + standard care	Qidaozhi Tongfu Recipe+standard care	14	②
Kang Hongyang [xv], 2014	78	21/18	16/23	1.18±1.15	1.37±1.62	Infant massage + standard care	Live Combined Bifidobacterium,Lactobacillus and Enterococcus Capsules +standard care	7~35	③
Zhang Yuxia [xvi], 2019	84	23/19	25/17	1.54±0.39	1.48±0.37	Infant massage + Medilac-Vita	Medilac-Vita	14	①
Cao Qiaohua [xvii],2017	70	16/19	18/17	1.40±0.13	1.43±0.10	Infant massage	Siliankang	14	①
Zhu Mei [xviii], 2012	116	32/28	30/26			Infant massage + Siliankang and standard care	Siliankang and standard care	30	①
Li Xiao[xix], 2018	80	23/17	22/18	6.69± 3.74	6.58± 3.88	Infant massage + Medilac-Vita and routine care	Medilac-Vita and routine care	5	①
Li Yuxia[xx], 2018	90	22/23	23/22	3.6±0.5	3.5±0.7	Infant massage	Xiaoer Kaiwei Zengshi Mixture, Oral	21	①⑤
Li Qianghua[xxi], 2008	86	24/22	22/18			Infant massage	Medilac-Vita, Oral	5~10	①
Yu Yuehao[xxii], 2013	80	21/19	18/22			Infant massage	Retention enema with kaiselu	3~6	①
Liu Liling[xxiii], 2017	100	31/19	29/21	3.02±0.82	2.92±0.85	Infant massage	Medilac-Vita, Oral	10	①

Note: Evaluation indicators: ① Efficacy; ② Stool form; ③Defecation frequency; ④ Defecation difficulty; ⑤ Score of constipation symptoms.

2. Search strategies: Four databases in Chinese and English language were searched, including: Pubmed, CNKI, WanFang and VIP. Since search strategies were the same for Chinese database, only search criteria for the representative Pubmed and CNKI were listed as follows.

The search model was as follows:

1.Pubmed 2021/3/20

#1 search:((((tuina[Title/Abstract]) OR (massage[Title/Abstract])) OR (manipulation [Title/Abstract])) OR (spinal manipulation[Title/Abstract])) OR (chiropractic[Title/Abstract]))

#2 search:(((children[Title/Abstract]) OR (infant[Title/Abstract])) OR(child[Title/Abstract]))

#3 search: *constipation[Title/Abstract]

#4 serach:"massage" [MeSH Terms]

#5 search:"chiropractic"[MeSH Terms]

#6 search:"spinal"[MeSH Terms]

#7 search:"child"[MeSH Terms]

#8search:"infant"[MeSH Terms]

#9search:"constipation"[MeSH Terms]

#10 search: #1 OR #2 OR #3 OR #4 OR #5 OR #6 OR #7 OR #8 OR #9

#11 search: ("2010/01/01"[Date-Publication]:"2020/03/20[Date-Publication])

2. CNKI 2021/3/20

#1 search:(((([SU=tuina) OR (SU=massage)) OR (SU=manipulation)) OR (SU=spinal manipulation)) OR (SU=chiropractic))

#2 search:(((SU=children)) OR (SU=infant)) OR(SU=child))

#3 search: *SU=constipation

#4 search: #1 OR #2 OR #3

#5 search: ("2010/01/01"[Date-Publication]:"2020/03/20[Date-Publication])

3. Sensitivity analysis

3.1 Stool form score

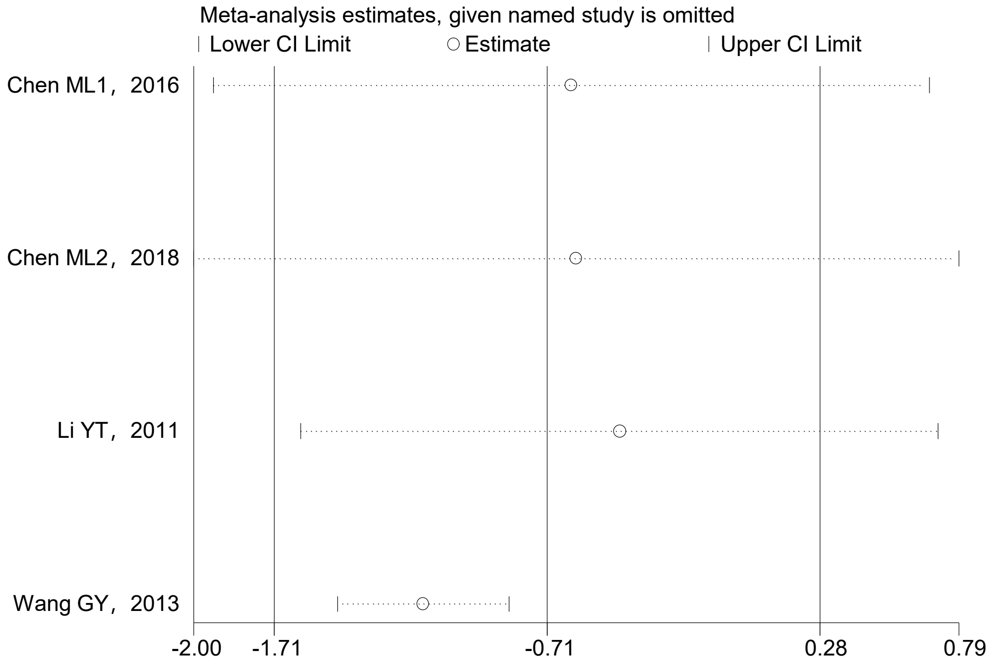

3.2 Defecation frequency

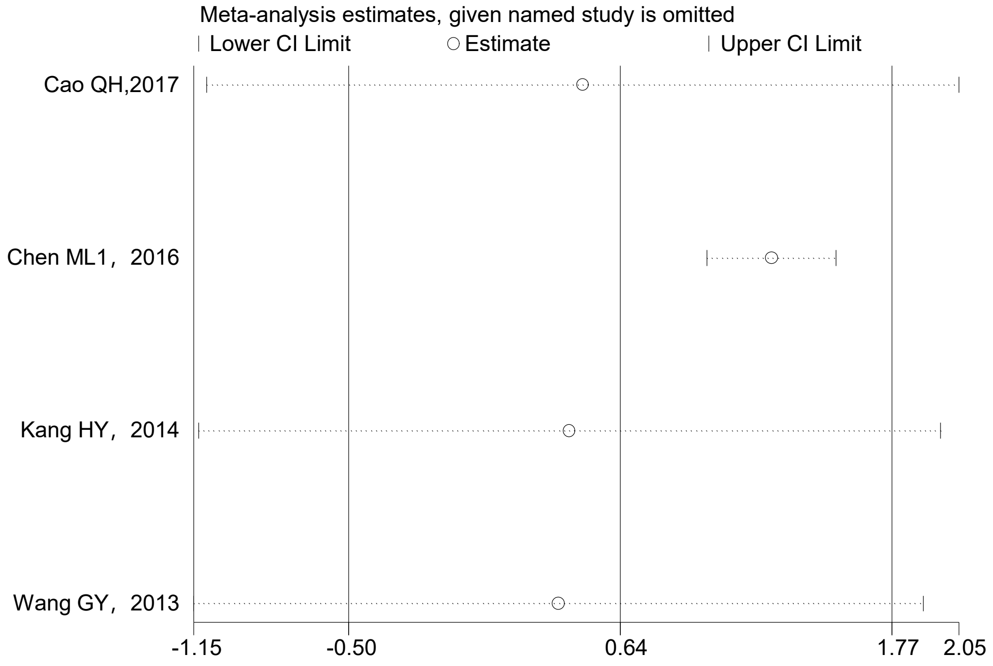

3.3 Constipation symptom score

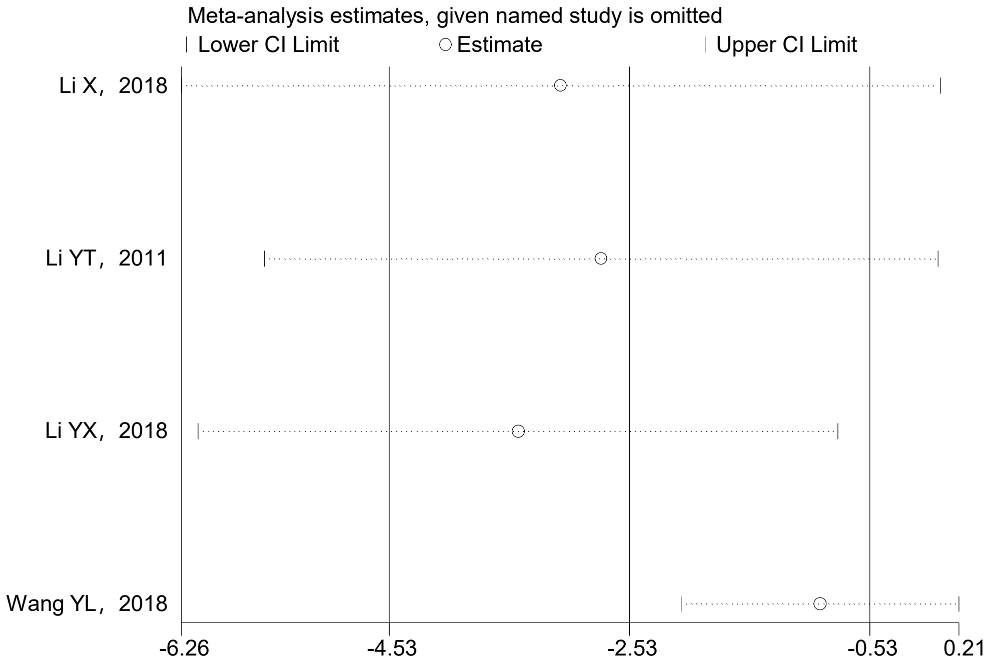

Conflict of Interest

Te authors declare that they have no competing interests, and due care has been taken to ensure the integrity of the work. Neither the entire paper nor any part of its content has been published or has been accepted elsewhere. It is not being submitted to any other journal. The authors have no other relevant affiliations or financial involvement with any organization or entity with a financial interest in or financial conflict with the subject matter or materials discussed in the manuscript apart from those disclosed.

Author Contributions

All authors have made substantive contributions to this study in regard to design and implementation. Z.L.,G.L. took part in the design of the study,performed the literature survey and drafted the manuscript. YW.M. took part Data Management implementation of the study. Z.L. was responsible for statistical analysis. Z.L. was responsible for the methodological design of study.L.L.&. Z.L. is the corresponding authors for the article. All the authors read and approved the final manuscript.

Acknowledgments

We gratefully acknowledged the cooperation of all research staff and participants.Te funding was supported by the National Natural Science Foundation of China (No.81503456), and the Tianjin enterprise science and technology commissioner project (No.18JCTPJC65500).
