# Supplementary figures and images for: Clinical Efficacy of Infantile Massage in the Treatment of Infant Functional Constipation: A Meta-Analysis
Source: Front Public Health. 2021 Jun 11;9:663581. doi: 10.3389/fpubh.2021.663581 (PMC8232057; doi:10.3389/fpubh.2021.663581)

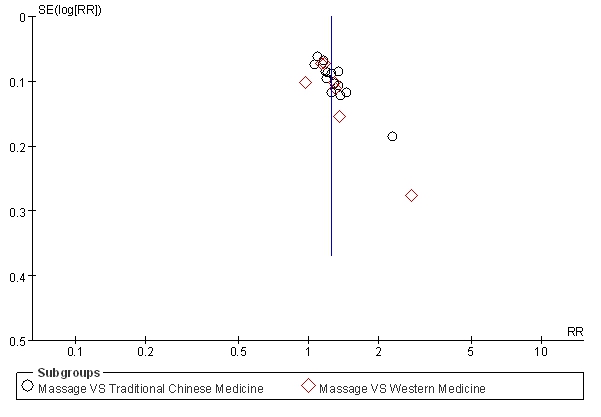

Supplement: Supplementary file 3 [file Image_1.JPEG]

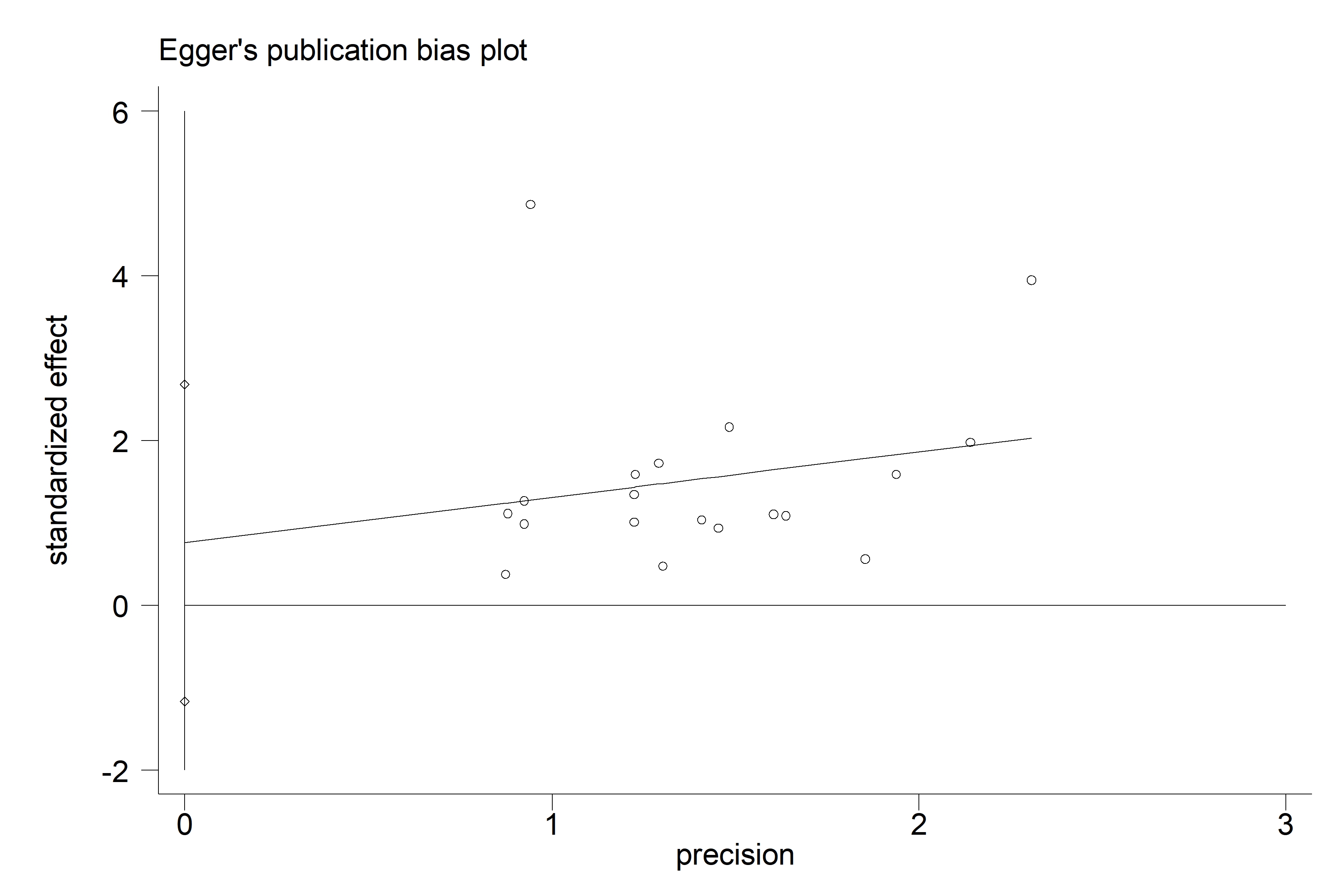

Supplement: Supplementary file 4 [file Image_2.JPEG]

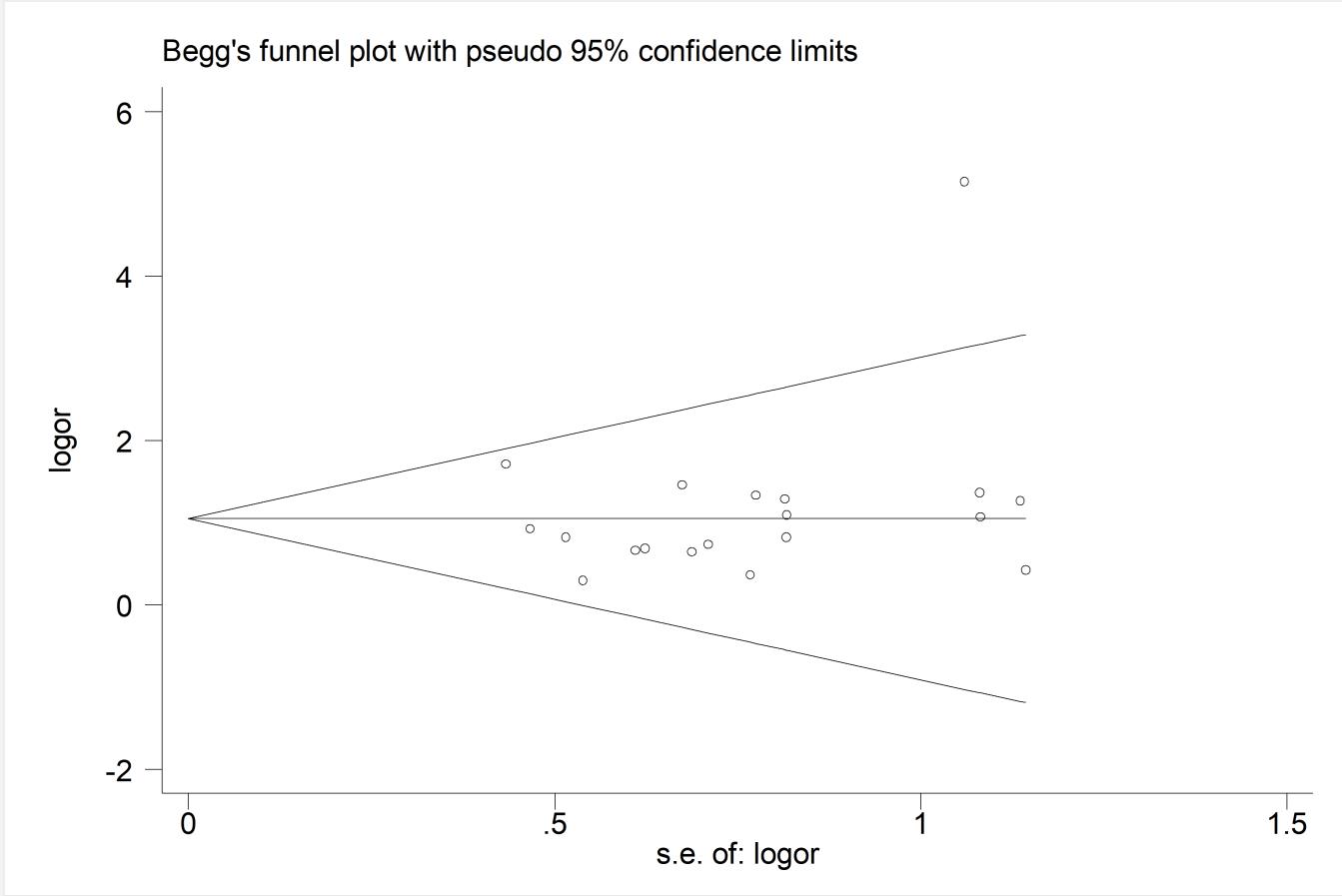

Supplement: Supplementary file 5 [file Image_3.JPEG]

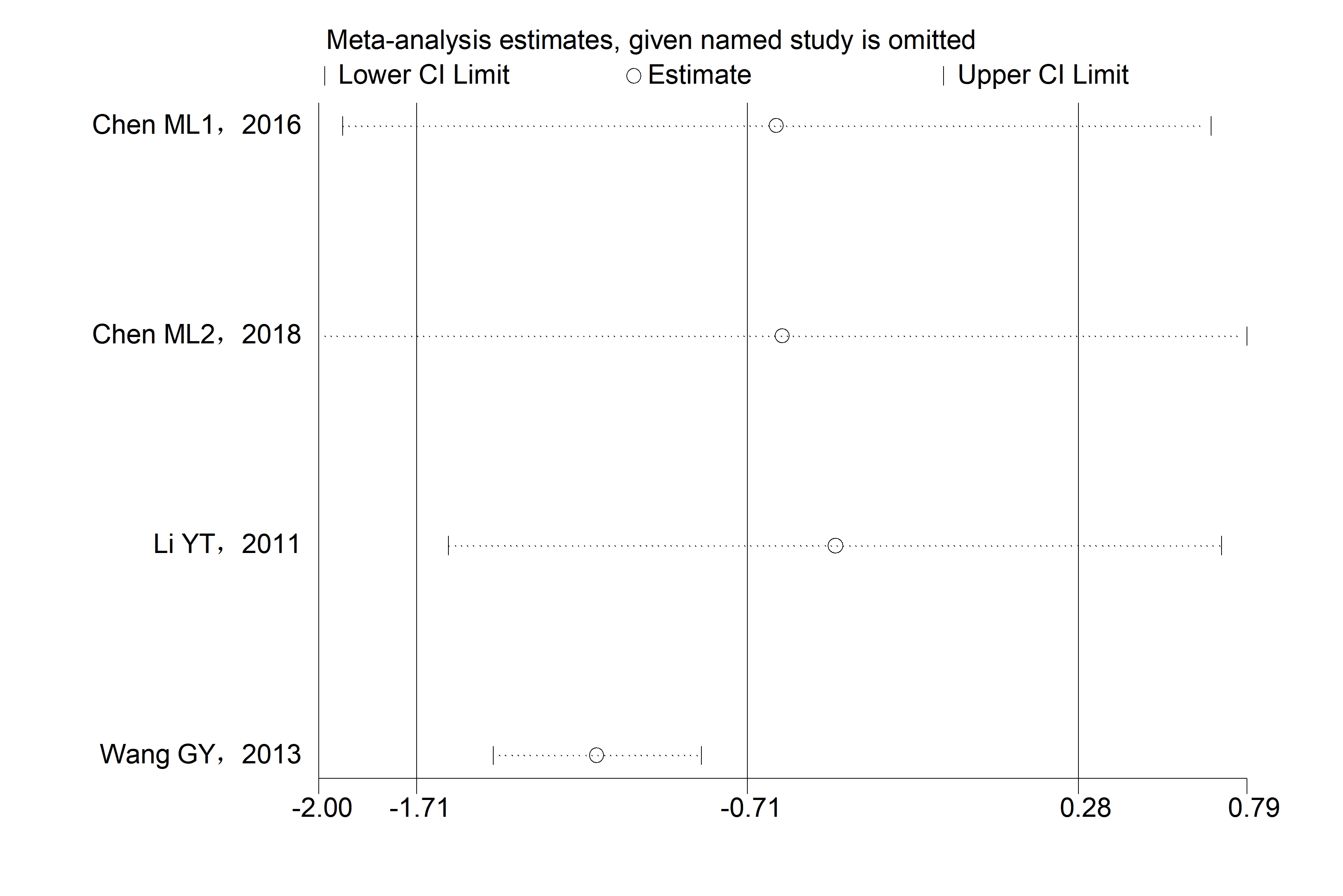

Supplement: Supplementary file 6 [file Image_4.JPEG]

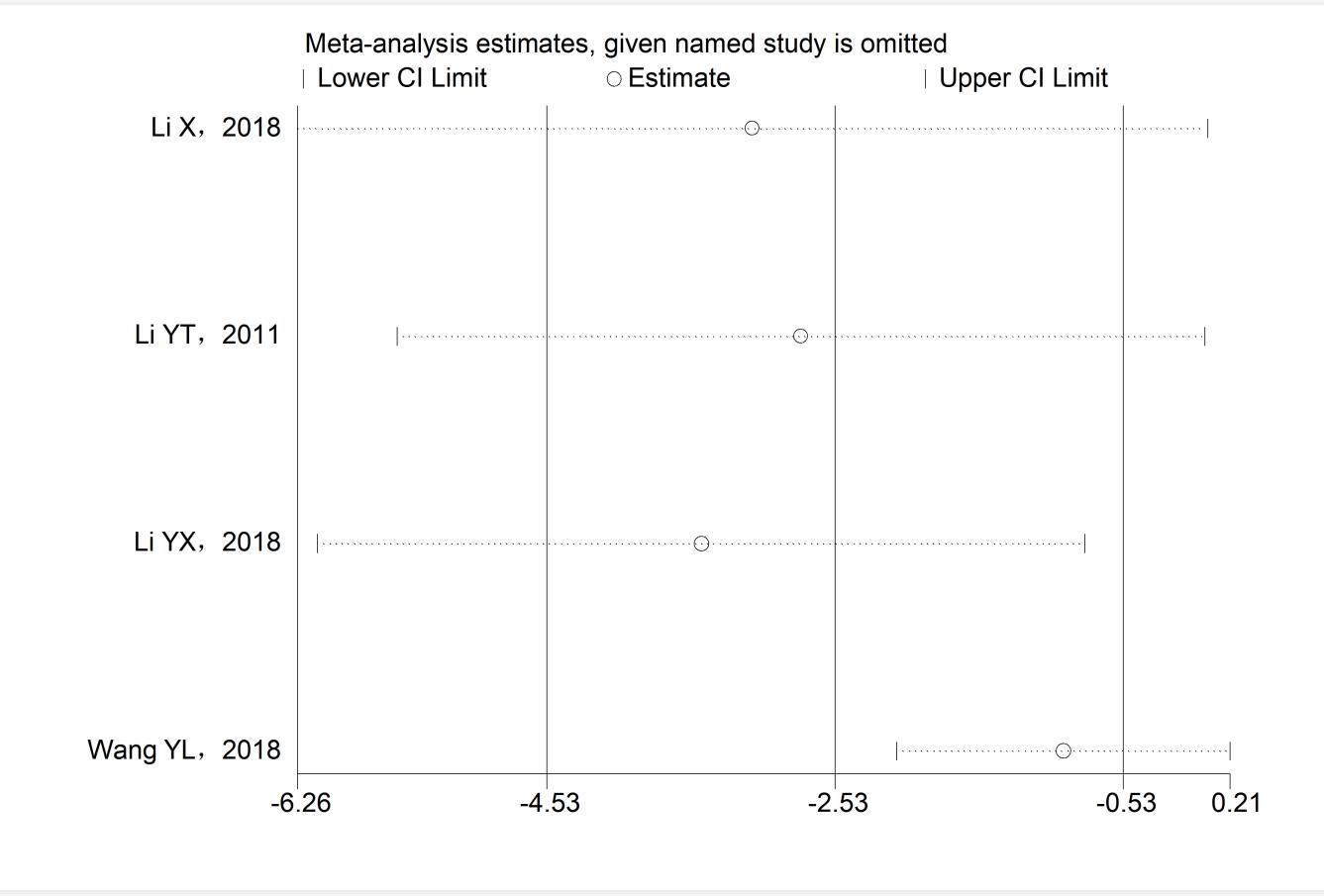

Supplement: Supplementary file 7 [file Image_5.JPEG]

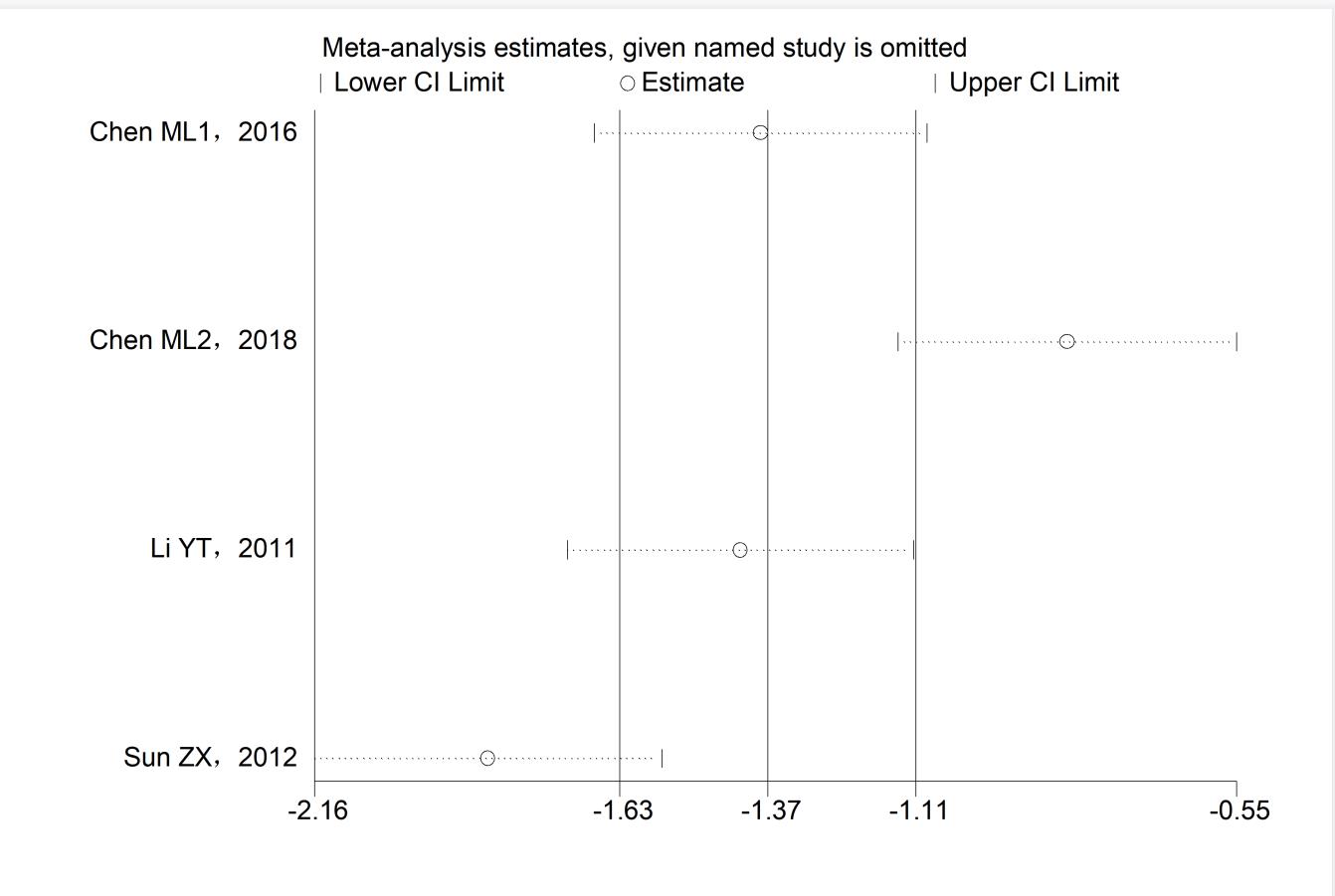

Supplement: Supplementary file 8 [file Image_6.JPEG]
